# Neuromuscular Electrical Stimulation to Maximize Hip Abductor Strength and Reduce Fall Risk in Older Veterans: Protocol for a Randomized Controlled Trial

**DOI:** 10.2196/68082

**Published:** 2025-05-01

**Authors:** Ben Friedman, Brock A Beamer, Jeffrey Beans, Vicki Gray, Gad Alon, Alice Ryan, Leslie I Katzel, John D Sorkin, Odessa Addison

**Affiliations:** 1 Department of Physical Therapy and Rehabilitation Science School of Medicine University of Maryland, Baltimore Baltimore, MD United States; 2 Baltimore Geriatric Research, Education, and Clinical Center VA Maryland Health Care System Baltimore, MD United States; 3 Division of Gerontology, Geriatrics and Palliative Medicine School of Medicine University of Maryland, Baltimore Baltimore, MD United States

**Keywords:** neuromuscular electrical stimulation, balance intervention, fall prevention, hip abductor strengthening, dynamic balance, veterans, muscle function, gait variability, multimodality balance intervention, fall prevention

## Abstract

**Background:**

Nearly half of all veterans are 65 years and older, and they have a higher prevalence of functional disabilities compared to the nonveteran population. Balance impairments resulting in injurious falls are a leading cause of morbidity and mortality in older adults. Instability or fear of falling can significantly reduce physical activity and social participation, even in the absence of falls. Dysmobility is a leading factor in long-care admissions, and therefore, maintenance of mobility throughout aging is crucial. Recent evidence indicates lower extremity muscle weakness as a key risk factor for falls, with lower limb muscle strength and quality being critical for balance recovery. The primary hip abductors, the gluteus maximus, medius, and minimus, are particularly essential for balance recovery.

**Objective:**

This study aims to test the hypothesis that adding neuromuscular electrical stimulation (NMES) to a multimodality balance intervention (MMBI) will yield greater reductions in fall risk and improvements in muscle and mobility function compared with MMBI alone.

**Methods:**

This randomized controlled trial will enroll 80 veterans aged 55 years and older at risk for falls (defined by a four-square step test [FSST] time >12 seconds, history of falls, or fear of falling). Participants will be randomized to receive either NMES + MMBI or MMBI alone. The 12-week outpatient center–based intervention will include 3 sessions per week, focusing on hip abductor strength, balance, and mobility. Assessments will occur at baseline, postintervention, and at 6- and 12-month follow-ups. Primary outcomes include fall risk and dynamic balance, measured by FSST and hip abductor strength using a Biodex dynamometer. Secondary outcomes will examine muscle composition through computed tomography (CT) scans and assess gait variability parameters.

**Results:**

This study was funded on January 1, 2022, with a data collection period from April 1, 2022, to December 31, 2026. As of March 2025, we have screened 100 potential participants and excluded 38. Out of the 61 participants enrolled to date, 21 have completed the 12-month follow-up, 32 have completed the 6-month follow-up, and 41 have completed the posttesting. A total of 4 participants are currently in the intervention phase; 1 has just completed the baseline testing, while 15 have been dropped from the study.

**Conclusions:**

This trial will be the first large, randomized controlled trial to evaluate NMES as an adjunct to an MMBI for fall prevention in older veterans. If successful, NMES combined with hip abductor strengthening and balance training could provide a low-cost, scalable solution to reduce falls, improve balance and mobility, and decrease health care costs related to falls in older adults. This study will address a critical gap in knowledge about the effectiveness of NMES in enhancing rehabilitation outcomes for fall prevention.

**Trial Registration:**

ClinicalTrials.gov NCT04969094; https://clinicaltrials.gov/study/NCT04969094

**International Registered Report Identifier (IRRID):**

DERR1-10.2196/68082

## Introduction

Recent census data place the share of veterans aged 65 years and older at nearly 50% of the total veteran population [1]. Injurious falls due to impaired balance function are a leading cause of morbidity and mortality among older adults [2]. Even in the absence of falling, older adults who are unstable or perceive they have an increased risk of falling reduce their levels of physical activity and social participation [3,4]. The ability to safely maintain mobility with aging is critical, as immobility is the leading cause of long-term care admissions [5].

While the causes of balance dysfunction and dysmobility are multifactorial, recent evidence demonstrates lower extremity muscle weakness as a main risk factor for both single and recurrent falls [6]. Numerous studies have reported lower limb muscle strength and quality are important predictors in the ability of older adults to recover from a loss of balance [7-10], and data from our laboratory further shows that the hip abductors—namely the gluteus maximus, medius, and minimus—are particularly important in recovery from balance perturbations [11-13]. Understanding the contributions of the hip abductors to balance and mobility, and finding targeted means to improve their function, opens important new treatment options for older adults with mobility limitations or who are at high risk of falls.

We have previously reported increased levels of intramuscular fat (IMAT) in the hip abductors of older adults who are at risk for falls [11,13]. Furthermore, it is well known that excessive levels of IMAT negatively affect skeletal muscle tissue [14,15]. Increased IMAT of the hip abductors is related to increased gait variability [11], decreased mobility and balance, and ultimately may be one potential contributor to increased risk for falls in older adults [11-13]. Both human [16] and animal studies [17], as well as our preliminary unpublished work, show that IMAT may impair the ability of the muscle to contract, ultimately impairing the ability to make significant improvements with exercise interventions [18,19].

Older adults suffer a loss of strength up to 3 times greater than the loss of muscle mass, suggesting the presence of intrinsic alterations in the force-generating capacity of skeletal muscle [20] One suggested mechanism of this decreased muscle quality is that older adults may suffer from an inability to fully activate muscle fibers and may, therefore, require augmentation to volitional resistance exercise to gain the full benefits [20]. While it is likely that multiple reasons for this impaired activation exist, IMAT has been identified as a potential contributor to impaired muscle activation [15,16,21]. Neuromuscular electrical stimulation (NMES) is an intervention that may improve muscle activation. While various forms of electrical stimulation have been applied to the gluteus medius during walking with the intent to improve gait [22,23], large sample, randomized controlled trials using electrical stimulation applied to the hip abductors to decrease falls have not been studied. Improving the composition and function of the primary hip abductors may ultimately result in improved balance and mobility function in older adults at risk for falls.

Understanding the contributions of the hip abductors to balance and mobility and finding targeted means to improve their response to exercise interventions opens important new treatment options for older adults with mobility limitations or who are at high fall risk. We propose to test the hypothesis that the addition of NMES applied to the primary hip abductors during a multimodality balance intervention (MMBI) will result in greater reductions in fall risk and larger improvements in muscle and mobility function than an MMBI alone.

## Methods

### Overview

**Figure 1:**
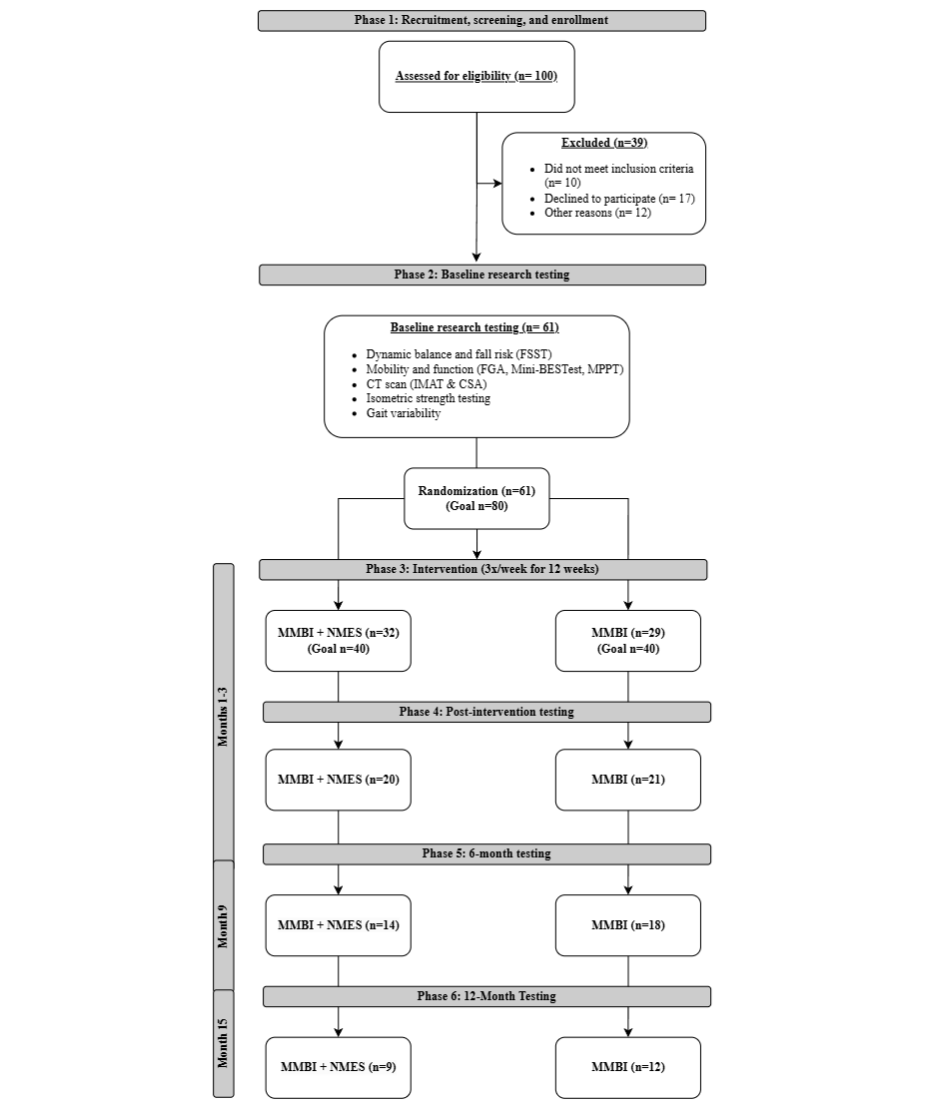
Experimental design. BEST: Balance Evaluation Systems Test; CSA: cross-sectional area; CT: computed tomography; FGA: Functional Gait Assessment; FSST: four-square step test; IMAT: intermuscular adipose tissue; MMBI: multimodality balance intervention; MPPT: Modified Physical Performance Test; NMES: neuromuscular electrical stimulation.

**Figure 2:**
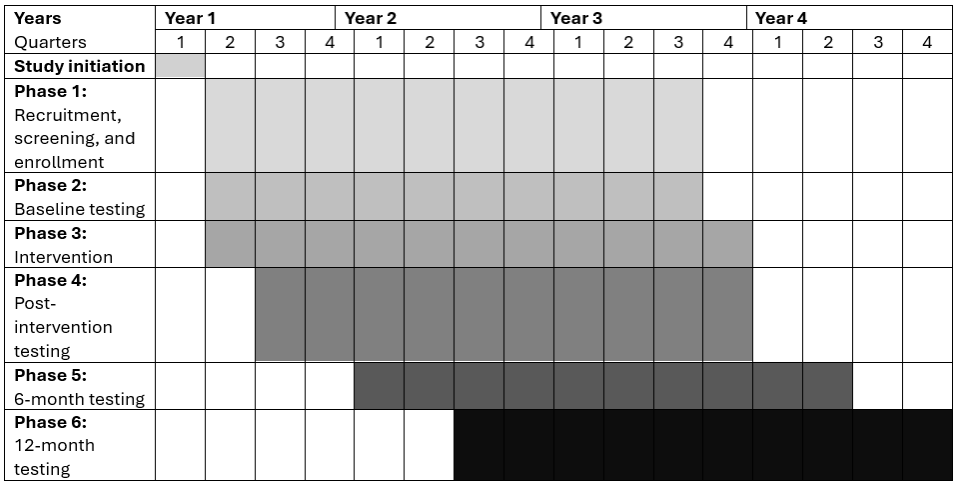
Study timeline.

This randomized control trial (trial registration: ClinicalTrials.gov NCT04969094) will examine the impact of 12 weeks (3 times per week) of NMES + an MMBI versus an MMBI alone on fall risk, balance, mobility, and hip abductor composition and strength in 80 (40/group) older veterans at risk for falls. Following completion of the 12-week outpatient center-based intervention, individuals will be provided with a written home exercise program, and we will reexamine subjects at 6 months and 1 year post intervention to assess retention of changes in hip abductor function, mobility, and fall risk. We will enroll veterans aged 55 years and older who are currently at risk for a fall (defined as a four-square step test [FSST] time >12 seconds [24], or a history of a previous fall in the last year, or a reported fear of falling). This study has 6 phases, completed over ~15 months per person. Figure 1 details the study design, and Figure 2 shows the study timeline. The study protocol follows the SPIRIT (Standard Protocol Items: Recommendations for Interventional Trials) guidelines [25]. Any protocol deviations, confidentiality breaches, or reportable adverse events will be reported to the respective institutional review board (IRB) and data safety monitoring board in accordance with local policies. In addition, the data safety monitoring board will conduct at least annual reviews of study materials and collected data to ensure integrity, security, and quality control.

### Setting

All testing and interventions will take place in a Veterans Affairs (VA)-based outpatient geriatrics exercise and rehabilitation center.

### Participants

An a priori power analysis was conducted for sample size estimation based on changes in the FSST and hip abductor strength in our nonrandomized pilot study. We estimate that 18-22 participants per group will be required to detect between group changes (effect size of 0.91-1.06; α=.05; β=.80) after 3 months of intervention for our primary outcomes of FSST and hip abductor strength. We aim to have 30 participants in each group complete the 3-month intervention and the 6- and 12-month retention testing phases. Accounting for an up to 25% dropout rate from enrollment through 12-month retention testing, we will randomize 40 individuals per group.

### Eligibility

Participants will be excluded if they have any of the following characteristics that impair their ability to safely participate in exercise or the NMES intervention: (1) high cardiovascular risk (such as poorly controlled hypertension of >160/100 mm Hg, symptomatic angina at rest or with exercise, or syncope in the last year without known resolution of cause), (2) require home oxygen, (3) contraindications to resistance training such as active proliferative retinopathy, (4) contraindications for NMES such as any implantable cardiac device, (5) dementia, and (6) other medical conditions that preclude patient participation in this study as per medical judgment of study team.

### Recruitment and Screening

Recruitment will focus on enrolling participants who are veterans. Participants will be recruited using the Department of Veterans Affairs Informatics and Computing Infrastructure and will be recruited from the Baltimore metropolitan area through media advertisement. Interested participants will be pre-screened through a standardized phone questionnaire. Individuals who pass the prescreening will be invited to our facility. After providing written informed consent and HIPAA (Health Insurance Portability and Accountability Act) authorization, they will undergo a history and physical examination conducted by the study’s medical personnel to ensure they (1) meet all inclusion and exclusion criteria and (2) can safely participate in the intervention.

### Randomization

After baseline testing, participants will be randomized 1:1 to either NMES + MMBI versus MMBI alone using computer-generated random numbers by the study biostatistician. We will use block randomization to stratify participants by age (55-64 years and aged 65 years and older). To address potential concerns of age after the completion of the study, we will also adjust any analysis with age as an independent factor. Group assignments will be placed in sealed envelopes. Upon recruitment and completion of baseline testing, the research coordinator will open the next envelope and record the group assignment. Only the biostatistician will have access to and knowledge of the randomization list.

### Intervention

#### Study Initiation

The intervention phase will last for 12 weeks with 3 sessions per week (for a maximum of 36 sessions). We will enroll individuals on a rolling admission basis. Admission to the study was originally planned to end at the end of year 3 to ensure that all individuals can be recruited, enrolled, and complete all study activities within a 4-year time frame. However, due to safety protocols related to COVID-19, initial enrollment was slower than originally anticipated. Now, enrollment is planned to continue for the first 3.5 years of the study.

#### The MMBI

The multimodality intervention is designed to increase lower extremity strength (with a focus on the hip abductors), improve balance, and decrease mobility deficits of older veterans at risk for falls. All individuals in the study, regardless of group allocation, will follow the same exercise protocol for the MMBI dynamic balance classes. Table 1 shows a detailed summary of the dynamic balance class, resistance training, and obstacle course components. Briefly, the dynamic balance class is designed to focus on common balance problems such as slow speed, foot clearance, directional changes, and obstacle negotiation. Exercises targeting these common conditions will include variations of functional movement patterns such as lunges, sit-to-stands, single-leg stance, and stepping tasks, individualized to the specific participant. Dual-task components, either cognitive or physical, will be added to one or more of the exercises to increase complexity. The resistance training component will include exercises that target the hip abductors, muscles of the lower extremity, and a core exercise. The obstacle course component will build on the skills developed in the dynamic balance class and apply them in a more varied and challenging environment. Vital signs will be assessed before and after each session to ensure safety. Over the 12 weeks of training, the intensity of exercise and the challenge to balance will gradually increase in difficulty. A trained and skilled instructor will lead each class, and 1-2 assistants will be present to assist with fall risk prevention. Classes will be capped at 10 participants per class for safety. The MMBI will occur in a supervised outpatient center-based setting. Participants are required to complete a minimum of 27 sessions. Those who are unable to complete the 27 sessions are not included in the final analysis. During the intervention phase, participants will be restricted from engaging in other forms of resistance training for the lower extremities, including receiving physical therapy services.

**Table 1:** Summary of the multimodality balance intervention.

Class portion	Duration (minutes)	Components
Dynamic balance class	30	Focused on common balance problems Includes variations on the following functional patterns: Lunge Sit to stand Single leg stance or range of motion Stepping task
Lower extremity resistance training	20	Exercises: Standing hip abduction^a^ Leg press Knee flexion Knee extension Calf raise Core activation Intensity: 60%-70% 1 repetition maximum Volume: 12 reps × 2 sets
Supervised obstacle course	10	Stepping over and around obstacles Walking on varying compliant surfaces Ascending and descending stairs Increasing gait speed

^a^Neuromuscular electrical stimulation (NMES) will be applied during this exercise for the NMES + MMBI (multimodality balance intervention) group.

#### MMBI + NMES

Individuals randomized to the NMES group will be asked to wear a portable NMES device applied to the hip abductors during the performance of the hip abduction strengthening exercise. We will place two 3” × 5” electrodes on each hip (4 total electrodes) to target the gluteus medius and minimus without the activation of the tensor fasciae lata (TFL). Table 2 shows a detailed description of the placement and stimulus parameters. Study staff will record the duration and NMES stimulus intensity in milliamps (mA) from the display of the stimulator screen during each session. As done in other studies of older adults, participants will initially be instructed to turn up the NMES intensity as high as tolerated [26]. After the first 30 seconds, participants will be encouraged to increase the intensity even higher if tolerated. This will be the only increase in intensity during a session and will be recorded as the intensity (mA) for the session. Repetitions of the hip abduction exercise will only be performed while the stimulation is active. We anticipate that the total active time will not exceed 4 minutes per side.

**Table 2:** Summary of neuromuscular electrical stimulation (NMES) parameters and placement.

Parameters	Settings
Amplitude (intensity)	As high as tolerable for the first 30 s Encourage to increase the intensity once, if tolerated
Frequency	50 Hz
Pulse width	300 µs
Ramp time	1 second up and down
On and off time	30 s on and 20 s off
Waveform	Symmetrical biphasic
Electrode placement	Proximal electrode just inferior to the iliac crest Distal electrode 0.5” below proximal, above the greater trochanter

#### Retention Phase

After the 12-week intervention period, participants from both groups will follow a home exercise program to be completed 3 times per week for 12 months. The exercises will mimic those from the resistance training component, including band-resisted standing hip abduction, knee extension, knee flexion, and seated pelvic tilts. In addition, participants will perform 3 exercises from the dynamic balance class: sit-to-stands, multidirectional weight shifts or lunges, and single-leg stance. Each exercise will be performed for 2 sets of 12 repetitions, except for the single-leg stance, which will be performed for 10 repetitions of 10 seconds bilaterally. Participants will receive instructions on proper forms and a detailed handout for reference. To enhance retention, a study staff member will call participants once per month during the prospective tracking phase. These calls will also address potential barriers to continued participation and plans for follow-up testing. For those who choose not to use the Annie App (Veterans Affairs) to track falls (described below), fall monitoring will be conducted through the scheduled monthly phone calls and mail-in postcard reporting.

### Outcome Measures

Outcome measures will be assessed at baseline, following the 12-week intervention, 6 months after completion of the intervention (~9 months from baseline testing), and 12 months after completion of the intervention (~15 months from baseline testing). Testing will generally be conducted over 3 visits. Table 3 shows the details of the testing process.

**Table 3:** Description of the research testing process.

Testing schedule	Description of testing
Timing	Tests performed at baseline will also be performed at 12 weeks and at the 6-month and 12-month intervals.
Visit 1	Dynamic balance and fall risk (FSST^a^) Mobility and function (FGA^b^, Mini-BESTest^c^, MPPT^d^)
Visit 2 (≥2 days later)	Gait variability
Visit 3 (≥2 days later)	Isometric strength testing Whole body DXA^e^ Spiral CT^f^ scan (IMAT^g^ and CSA^h^)

^a^FSST: four-square step test.

^b^FGA: Functional Gait Assessment.

^c^Mini-BESTest: Mini Balance Evaluation Systems Test.

^d^MPPT: Modified Physical Performance Test.

^e^DXA: dual-energy x-ray absorptiometry.

^f^CT: computed tomography.

^g^IMAT: intramuscular adipose tissue.

^h^CSA: cross-sectional area.

#### Primary Outcome (Aim 1)

The FSST is a test of dynamic balance and lateral mobility and will be used to assess fall risk and dynamic balance [24]. The FSST mimics real-life scenarios requiring multidirectional movement and balance and demonstrates excellent inter-rater and retest reliability in older adults [24]. Subjects will be timed as they step over 4 canes set up in a cross on the floor. The test will be performed twice, and the best time of the 2 tests will be used. As described in the original test, individuals will be able to use a cane if needed [24].

#### Secondary Outcomes (Aim 1)

The Functional Gait Assessment (FGA) and Mini-BESTest will also be used as measures of mobility and balance. The FGA is a 10-item clinical gait test that is based on the dynamic gait index [27]. It tests higher-level dynamic gait activities that are part of daily functional mobility, such as turning safely and ambulating backward [27]. Total scores range from 0 to 30, and scores below 22 are predictive of future falls [27]. The mini-BESTest identifies 6 different balance control systems, including anticipatory postural adjustments, reactive postural control, and dynamic gait [28]. It is a 14-item test with a total possible score of 28 points [28].

The Modified Physical Performance Test (MPPT) will be used to examine physical function. The MPPT is a 9-item standardized test used to identify frailty and mobility dysfunction in older individuals [29]. Individuals are scored on the time it takes to complete tasks such as donning and doffing a coat, picking a penny from the ground, and ascending the stairs. These scores are then summed (the maximum value of the sum is 36). Scores less than 32 indicate at least mild frailty [29].

#### Primary Outcome (Aim 2)

Hip Abductor Strength: Hip abductor muscle strength, operationally defined as the peak maximal voluntary isometric contraction (MVIC) of hip abductor torque, will be measured using a Biodex System 4 Pro Dynamometer. The position will be standardized and maintained during all testing time points. Hip abduction strength will be tested in the side-lying position at 10 degrees of hip abduction [30] to minimize the influence of hip abductor strength of the contralateral leg as compared with a standing position [31]. We will assess, as a secondary outcome measure of lower extremity strength, maximal voluntary knee extension strength with isometric knee extension in a seated position at 60 degrees of flexion following Biodex protocols.

#### Secondary Outcomes (Aim 2)

##### Computed Tomography Scans and Muscle Composition Assessment

A spiral computed tomography (CT) scan (starting at the L2-L3 interspace and extending to the patella) will be performed at baseline, after the 12 weeks of training, and at the 6- and 12-month postexercise intervention follow-ups. The analysis will focus on the primary hip abductors (gluteus medius and minimus), the tensor fascia lata, and the quadriceps. As we have previously published [11,13], the cross-sectional area (cm^2^) of high-density lean tissue and low-density lean tissue content will be determined by manual segmentation using Medical Image Processing, Analysis, and Visualization (version 11.3.3; National Institutes of Health) analysis software. Research staff performing the image analysis will be blinded to the participant group allocation. CT data will be expressed as a cross-sectional area of tissue for each muscle of interest (cm^2^). High-density lean tissue will be defined using Hounsfield units between 30 and 100, and low-density lean tissue as 0 to 29 Hounsfield units. Muscle composition will be determined by identifying the percentage of high-density and low-density lean tissues in both the primary (gluteus medius and gluteus minimus) and secondary (tensor fascia lata) hip abductor muscles. High-density lean and low-density tissues will be normalized for the respective muscle’s size by calculating a percentage of each measure relative to the total muscle cross-sectional area. As in previous studies, high-density lean tissue will be used as a measure of lean tissue, and low-density lean tissue will be used as a measure of IMAT [32].

##### Gait Variability

As a secondary outcome and preliminary data collection for future studies, we will assess measures of gait variability during walking utilizing kinematic recordings. We will use the Kinesis (Linus Health Technologies), a small, commercially available, portable sensor with a triaxial gyroscope and accelerometer that is worn below the knee. The Kinesis measures multiple gait variability parameters (stride, stance, swing and step times, single and double support times, stride velocity, and length) as coefficients of variation over the course of the walking trial or gait cycle, expressed as a percentage [33].

##### Responders and Nonresponders

As an additional outcome and preliminary data collection for future studies, we will examine those who improve (responders) and those who do not (nonresponders) in each group. We will define a responder as someone who improves hip abduction torque by 20% or greater. After identifying responders and nonresponders in both groups, we will compare their baseline hip abductor muscle composition (through CT scans) and muscle quality (force/cross-sectional area) to examine potential associations with responsiveness to the intervention. In the MMBI + NMES group, we will also assess stimulation dosage records.

Individuals will return for retention testing 6 and 12 months after the completion of the exercise interventions. Testing will occur over 2 visits and will include all tests done at baseline and post testing. We will also prospectively track the frequency of falls for 12 months following the completion of the training. Participants will be asked to use the Annie App, a VA-supported SMS text messaging app that uses text messaging to track weekly falls. We will familiarize veterans with the use of the app during their last week of training. If participants are not comfortable using the VA Annie app, they will be given the option to return monthly postcards reporting any falls over the last month.

### Data Analysis and Plan

For our primary and secondary outcomes, we will use repeated measures ANOVA (Statistical Analysis Software [SAS] MIXED Procedure) to compare values of our outcome variables at baseline, following the 12 weeks of intervention, and 6 and 12 months following completion of exercise: outcome=group + time + group × time. If there is an imbalance between the groups at baseline, the out-of-balance variable will be added as an additional independent variable to the model. We will use Corrected Akaike Information Criterion, a variation of Akaike’s Information Criteria, to select the correlation structure (unstructured, compound symmetry, autoregressive lag 1) that best accounts for the serial autocorrelation of repeated measures from the same subject. We will use per-planned contrasts (implemented using estimate statements) to compare the changes (3 months minus baseline; and 6 and 12 months minus baseline) in our 2 groups. We will use Poisson regression (SAS GENMOD Procedure) to compare the number of falls in our two groups over the course of the 1-year follow-up. Our analyses will be 2-tailed and will be performed following the principle of intention-to-treat. Missing data will be imputed using multiple imputation, which provides unbiased estimates of whether the pattern of missing data is missing completely at random or at random. If the pattern of missing data is nonignorable missing, accounting for missing values is complex and there is no fully satisfactory method for preventing bias other than modeling the mechanism that resulted in missing values which most often cannot practically be done, and in this case, multiple imputations is no worse than the usual approach of dropping subjects with missing data. Before accepting the results of the model, we will make sure the results conform to the assumptions of the model (eg, reviewing residual plots), and we will make sure that no observation has undue influence on the estimates produced by the model (eg, Cook D). We will conduct exploratory analyses to examine relationships among variables and their changes in response to the intervention. We will examine, in separate models, the association of hip abductor muscle properties including (1) peak MVIC as determined with the biodex, (2) cross-sectional area, (3) composition (% of intramuscular fat determined from CT), and (4) activation during gait as determined by electromyography as predictors of 5 outcome measures (1) FSST, (2) mini-BESTest, (3) functional gait assessment, (4) modified physical performance test, and (5) stride time variability. We will test the associations (1) at baseline and (2) we will examine the association between the change in the predictor variables and the change in the outcome variables. In addition, we will examine the association between the changes in baseline variables and fall rate, including the fall rate assessed during 1 year of follow-up post intervention, and the number of fall-free weeks. Finally, we will assess the association between the changes in predictor variables during the intervention and subsequent fall rates. This will provide a wealth of information on the interrelationship between hip abductors, balance, mobility, and fall risk.

### Data Management

Participants will be assigned a unique study ID stored in a password-protected database on a VA server that has a level and scope of security that equals or exceeds that established by the HIPAA Security Rules. Data collected during the study that is not captured electronically will be entered and stored in the VA REDCap (Research Electronic Data Capture; Vanderbilt University) system. Participants’ charts will be held in a locked room inside a locked cabinet. Access to individual participant numbers or personal identifiers will be limited to research team members who need these data to perform their roles in this study.

### Participant Safety and Minimizing Potential Risk

While we anticipate that there may be some risks associated with participating in this research program, we have attempted to minimize them. Subjects will undergo procedures that involve a mild-to-moderate degree of risk, including exercise and balance training and testing, strength assessments, and muscle composition assessments, including DXA and CT scans. Completion of exercise and balance training and testing is associated with the risk of falls and cardiovascular complications such as chest pain, heart attack, or sudden death, and complications related to stress and strains of muscles or twisted ankles. The risk of falling is comparable to the participants’ everyday lives, as the training is designed to use elements of everyday activities. We anticipate that some individuals may fall during the intervention, but the risk of falls and injury will be minimized by the supervision of all activities by trained study personnel. Furthermore, the risk of cardiovascular complications is minimal. The American Heart Association consensus statement on exercise standards estimates that the acute risk of sudden cardiac arrest during exercise training in participants with known cardiac disease is approximately 1 event per 60,000 hours of aerobic exercise [34]. The risk of exercise training is greater at higher exercise intensities. This risk is offset by prescreening participants with medical evaluations before exercise. There is also some risk for muscle soreness with the use of strength training as well as NMES. This will be minimized through the delivery of the intervention by trained study staff and proper education with all activities.

The risks of muscle composition assessments with DXA and CT scans include the risk of radiation exposure. While any dose of radiation could be potentially harmful, the radiation-related risks associated with the measurement of muscle composition are well within the established dosimetry of radiation guidelines for research participants, and radiation safety protocols will be maintained for all participants.

### Ethical Considerations

This study has been approved by the University of Maryland School of Medicine IRB (HP-00110719) and the Baltimore VA Medical Center Research and Development Committee in accordance with the ethical standards of responsible committees on human experimentation and with the Helsinki Declaration. Informed consent will be obtained from each participant before any study procedures, and they will be allowed to opt out at any point. Any adverse, notable, or unexpected events will be reported to an independent, internal Data Safety Monitoring Board. The University of Maryland IRB and the VA Maryland Health Care System Research and Development Committees require a data and safety monitoring plan for all studies classified as greater than minimal risk. This study meets the regulatory criteria for a greater than minimal risk study. As it is supported by the Baltimore VA Medical Center Geriatric Research and Education Clinical Center, per internal policies, it will undergo biannual review by the Baltimore VA Medical Center Geriatric Research and Education Clinical Center’s internal data safety monitoring board. The board's composition and standard operating procedures have been approved by the University of Maryland IRB. Its primary focus is safety, particularly monitoring expected and unexpected adverse events directly attributable to study participation (ie, falls on the treadmill).

### Dissemination

Results from this study will be available to health care professionals and the public through the National Library of Medicine PubMed Central website within 1 year after the date of publication. In addition, study findings will also be presented at scientific meetings. The study investigators will be solely responsible for drafting all publications and will not engage professional writers.

## Results

### Overview

This study was funded on January 1, 2022, with a data collection period from April 1, 2022, to December 31, 2026. As of March 2025, we have screened 100 potential participants and excluded 38. Of the 61 participants enrolled to date, 21 have completed the 12-month follow-up, 32 have completed the 6-month follow-up, and 41 have completed the posttesting. A total of 4 participants are currently in the intervention phase; 1 has just completed the baseline testing, while 15 have been dropped from the study (Figure 1).

### Expected Outcomes

Based on our preliminary data showing that the addition of NMES to the MMBI program results in greater improvements in the FSST and hip abduction strength, we anticipate that those in the NMES + MMBI group will make significantly greater improvements in all measures during the 12-week intervention compared with the MMBI-only group. As noted above, we anticipate that a small number of individuals will make no improvement following the intervention (nonresponders). We expect that we will have fewer nonresponders in the NMES + MMBI group compared with the matched MMBI group due to the superimposed muscle activation provided by the addition of NMES. We further hypothesize that the nonresponders in the MMBI-only group will exhibit increased IMAT and lower torque of their hip abductors at baseline, as the presence of IMAT is thought to contribute to impaired muscle activation [15,16,21]. While we anticipate that NMES will primarily maximize changes in the gluteus medius and gluteus minimus (the primary hip abductors), we will also examine how changes in these muscles, along with the tensor fasciae lata, contribute to changes in fall risk and strength. If changes in the TFL are advantageous, this information can be used to design a future trial targeting the use of the TFL to compensate for maladaptive changes in the primary hip abductors. We also anticipate that the NMES + MMBI group will demonstrate greater retention of the positive changes from baseline at both the 6- and 12-month timepoints following the cessation of the program and fewer falls due to improvements in the muscle quality of the primary hip abductors.

## Discussion

### Principal Findings

We anticipate that this VA Merit award will establish NMES as an efficacious therapy that can be used in conjunction with hip abductor strengthening and balance training programs for fall prevention. This comprehensive and augmented training approach has the potential to change existing treatment paradigms for the management of falls in older adults. NMES is a low-cost intervention that can be easily disseminated with the aim of reducing disability and health care costs related to the consequences of falls in older adults. While other studies have investigated the use of NMES in older adults, the majority have focused on its application to the more distal muscles of the lower extremities, such as the quadriceps or the plantar flexors [35]. The outcome measures in these small (often less than 20 people per intervention) studies [26,36,37] have been strength or static balance measures, but do not examine changes to balance and fall risk as they relate to the hip abductor alterations. Instead, NMES was frequently limited to unilateral application to measure changes in gait mechanics.[26] Also, these studies did not target older adults [36,38,39]. Our study will be among the first, large RCTs to examine the combined use of NMES on the hip abductors in conjunction with a multimodality balance intervention designed to improve balance and mobility and thereby reduce falls in older veterans.

Our study will use CT imaging to evaluate changes in muscle cross-sectional area and quality (ie, composition) posttraining. The use of CT imaging will allow us to examine changes in both the primary (gluteus minimus and gluteus medius) as well as the secondary (tensor fascia lata) hip abductors. Imaging the primary and secondary hip abductors will allow us to examine the changes and relative contributions of changes in both the primary and secondary hip abductors to mobility and fall risk in older veterans.

Understanding the contributions of the hip abductors to balance and mobility and finding targeted means to improve their function opens important new treatment options for older adults who are at a high fall risk. This study will address an important knowledge gap about the relative contribution of the primary and secondary hip abductors to dysmobility and fall risk. In previous studies examining the effects of resistance training on balance, less than 30% included any resistance training of the hip abductors [40] and no studies examined the hip abductors as a primary outcome measure, or used muscle imaging techniques such as CT scans to examine changes in both the primary and secondary hip abductors. Given our previous work demonstrating the importance of the hip abductors to balance recovery [11] and stepping [13], our study is a novel and important outcome in this population of older veterans. Finally, following the changes in both muscle composition and fall risk at 6 and 12 months after the end of training will allow us to evaluate the durability of the intervention to convey meaningful long-term benefits of the program.

### Limitations

Despite the numerous strengths of this study, it has several potential limitations. Our target population includes adults aged 55 years and older who are at risk of falls. This may confound our findings’ generalizability to other studies, as it is a younger age than many fall studies that traditionally only recruit older adults aged 65 years and older. We believe this traditional approach is suboptimal as falls are not just a problem of advanced age, as demonstrated by the earlier sharp increase in the prevalence of falls starting in middle age [41]. Excluding individuals 65 years and younger who are at high risk for falling (those who have previously had a fall, or those who demonstrate balance abnormalities) may prevent the opportunity for early intervention in an “at-risk” population [41]. Therefore, we intentionally decided to include a younger population in our study than the one traditionally used.

Another potential limitation of this study is the anticipated predominance of male participants. So far, we have enrolled 61 participants, consisting of 50 (82%) men and 11 (18%) women, which differs significantly from the general population, where 44.6% of individuals aged 65 years and older are male and 55.4% are female [42]. However, census data show that 96% of veterans in this age group are male [1], suggesting that our cohort may more accurately represent the veteran population. The inclusion of adults aged 55 years and older may explain the higher proportion of female participants in our study.

Our study does not include any “sham” treatment for NMES. The inclusion of a sham treatment with NMES on the hip abductors was considered. However, even low currents may have a confounding effect, and we therefore chose not to include a sham treatment. Finally, there are multiple other outcomes that could be evaluated, such as biomechanics. We have purposefully chosen our primary outcomes for this grant to be clinically meaningful, with an interest in hastening translation to patient care. Though we hope this study will inspire future biomechanistic studies, the inclusion of such measures is outside the scope of this project. Should NMES indeed enhance the beneficial effects of MMBI, there will be a strong rationale to examine the underlying biomechanical changes further.

### Future Directions

Our analysis of the responders and nonresponders will provide important information for future studies, as it may help us to identify a subpopulation of individuals that may benefit most from the application of NMES. Our collection of the NMES stimulation intensity (mA) used and the number of sessions attended will enable us to evaluate a dose response of NMES and changes as our primary outcomes. This information could be used to design a future trial that examines these variables to optimize the application. As previously described, we will also examine the contribution of changes in both the gluteus medius and gluteus minimus and the tensor fasciae lata to changes in fall risk and strength. This may lead to future trials targeting the TFL to compensate for maladaptive changes of the primary hip abductors if our analysis reflects that it is indeed advantageous. Thus, the proposed research from this study will not only provide valuable information about the use of NMES to improve hip abductors and reduce fall risk but also provide multiple future research directions.

### Conclusions

This clinical trial seeks to provide evidence from the first large, randomized control trial to test the hypothesis that NMES applied to the primary hip abductors, in addition to a multimodality balance intervention, is an efficacious adjunct to traditional rehabilitation to improve balance, mobility, and function in older veterans at risk for falls. If proven effective, this low-cost adjunct to resistance exercise can be easily transitioned into clinical practice to better reduce disability and defer health care costs related to falls in older adults.

## Appendix

Multimedia Appendix 1 Peer review from the United States Department of Veterans Affairs, Musculoskeletal Health and Function, Rehabilitation Research and Development Parent IRG.

## Abbreviations

CT: computed tomography
FGA: Functional Gait Assessment
FSST: four-square step test
HIPAA: Health Insurance Portability and Accountability Act
IMAT: intramuscular adipose tissue
IRB: institutional review board
mA: milliamps
MMBI: multimodality balance intervention
MPPT: Modified Physical Performance Test
MVIC: maximum voluntary isometric contraction
NMES: neuromuscular electrical stimulation
REDCap: Research Electronic Data Capture
SAS: Statistical Analysis Software
SPIRIT: Standard Protocol Items: Recommendations for Interventional Trials
TFL: tensor fasciae lata
VA: Veterans Affairs

## Data Availability

The datasets generated and analyzed during this study will not be publicly available due to confidentiality requirements and institutional policies of the Department of Veterans Affairs, but they will be available from the corresponding author upon reasonable request. A deidentified, anonymized dataset will be created and shared under a data use agreement, which will limit data use and prohibit any attempts to identify or reidentify individuals. Model consent forms and other related documentation given to participants will be uploaded to ClinicalTrials.gov as required.

## References

[1] Bureau UC Aging Veterans: America's Veterans in Later Life. Census.gov.

[2] Saveman B, Björnstig Ulf (2011). Unintentional injuries among older adults in northern Sweden--a one-year population-based study. Scand J Caring Sci.

[3] Chung MC, McKee KJ, Austin C, Barkby H, Brown H, Cash S, Ellingford J, Hanger L, Pais T (2009). Posttraumatic stress disorder in older people after a fall. Int J Geriatr Psychiatry.

[4] Martin FC, Hart D, Spector T, Doyle DV, Harari D (2005). Fear of falling limiting activity in young-old women is associated with reduced functional mobility rather than psychological factors. Age Ageing.

[5] Gaugler JE, Duval S, Anderson KA, Kane RL (2007). Predicting nursing home admission in the U.S: A meta-analysis. BMC Geriatr.

[6] Moreland JD, Richardson JA, Goldsmith CH, Clase CM (2004). Muscle weakness and falls in older adults: a systematic review and meta-analysis. J Am Geriatr Soc.

[7] Graham DF, Carty CP, Lloyd DG, Barrett RS (2015). Biomechanical predictors of maximal balance recovery performance amongst community-dwelling older adults. Exp Gerontol.

[8] Pijnappels M, van der Burg PJCE, Reeves ND, van Dieën JH (2008). Identification of elderly fallers by muscle strength measures. Eur J Appl Physiol.

[9] Addison O, Young P, Inacio M, Bair W, Prettyman M, Beamer B, Ryan A, Rogers M (2014). Hip but not thigh intramuscular adipose tissue is associated with poor balance and increased temporal gait variability in older adults. Curr Aging Sci.

[10] Inacio M, Ryan AS, Bair W, Prettyman M, Beamer BA, Rogers MW (2014). Gluteal muscle composition differentiates fallers from non-fallers in community dwelling older adults. BMC Geriatr.

[11] Lockhart TE, Smith JL, Woldstad JC (2005). Effects of aging on the biomechanics of slips and falls. Hum Factors.

[12] Pijnappels M, Reeves ND, Maganaris CN, van Dieën JH (2008). Tripping without falling; lower limb strength, a limitation for balance recovery and a target for training in the elderly. J Electromyogr Kinesiol.

[13] Addison O, Inacio M, Bair W, Beamer BA, Ryan AS, Rogers MW (2017). Role of hip abductor muscle composition and torque in protective stepping for lateral balance recovery in older adults. Arch Phys Med Rehabil.

[14] Addison O, Marcus RL, Lastayo PC, Ryan AS (2014). Intermuscular fat: a review of the consequences and causes. Int J Endocrinol.

[15] Sachs S, Zarini S, Kahn DE, Harrison KA, Perreault L, Phang T, Newsom SA, Strauss A, Kerege A, Schoen JA, Bessesen DH, Schwarzmayr T, Graf E, Lutter D, Krumsiek J, Hofmann SM, Bergman BC (2019). Intermuscular adipose tissue directly modulates skeletal muscle insulin sensitivity in humans. Am J Physiol Endocrinol Metab.

[16] Yoshida Y, Marcus RL, Lastayo PC (2012). Intramuscular adipose tissue and central activation in older adults. Muscle Nerve.

[17] Biltz NK, Collins KH, Shen KC, Schwartz K, Harris CA, Meyer GA (2020). Infiltration of intramuscular adipose tissue impairs skeletal muscle contraction. J Physiol.

[18] Marcus RL, Addison O, LaStayo PC (2013). Intramuscular adipose tissue attenuates gains in muscle quality in older adults at high risk for falling. A brief report. J Nutr Health Aging.

[19] Correa-de-Araujo R, Harris-Love MO, Miljkovic I, Fragala MS, Anthony BW, Manini TM (2017). The need for standardized assessment of muscle quality in skeletal muscle function deficit and other aging-related muscle dysfunctions: A symposium report. Front Physiol.

[20] Goodpaster BH, Park SW, Harris TB, Kritchevsky SB, Nevitt M, Schwartz AV, Simonsick EM, Tylavsky FA, Visser M, Newman AB (2006). The loss of skeletal muscle strength, mass, and quality in older adults: the health, aging and body composition study. J Gerontol A Biol Sci Med Sci.

[21] Correa-de-Araujo R, Addison O, Miljkovic I, Goodpaster BH, Bergman BC, Clark RV, Elena JW, Esser KA, Ferrucci L, Harris-Love MO, Kritchevsky SB, Lorbergs A, Shepherd JA, Shulman GI, Rosen CJ (2020). Myosteatosis in the context of skeletal muscle function deficit: An interdisciplinary workshop at the national institute on aging. Front Physiol.

[22] Al-Abdulwahab SS, Al-Khatrawi WM (2009). Neuromuscular electrical stimulation of the gluteus medius improves the gait of children with cerebral palsy. NeuroRehabilitation.

[23] Mooney JA, Rose J (2019). A scoping review of neuromuscular electrical stimulation to improve gait in cerebral palsy: The arc of progress and future strategies. Front Neurol.

[24] Dite W, Temple VA (2002). A clinical test of stepping and change of direction to identify multiple falling older adults. Arch Phys Med Rehabil.

[25] Chan A, Tetzlaff JM, Gøtzsche PC, Altman DG, Mann H, Berlin JA, Dickersin K, Hróbjartsson A, Schulz KF, Parulekar WR, Krleza-Jeric K, Laupacis A, Moher D (2013). SPIRIT 2013 Explanation and elaboration: Guidance for protocols of clinical trials. BMJ.

[26] Nussbaum EL, Houghton P, Anthony J, Rennie S, Shay BL, Hoens AM (2017). Neuromuscular electrical stimulation for treatment of muscle impairment: Critical review and recommendations for clinical practice. Physiother Can.

[27] Wrisley DM, Marchetti GF, Kuharsky DK, Whitney SL (2004). Reliability, internal consistency, and validity of data obtained with the functional gait assessment. Phys Ther.

[28] Anson E, Thompson E, Ma L, Jeka J (2019). Reliability and fall risk detection for the BESTest and Mini-BESTest in older adults. J Geriatr Phys Ther.

[29] Brown M, Sinacore DR, Binder EF, Kohrt WM (2000). Physical and performance measures for the identification of mild to moderate frailty. J Gerontol A Biol Sci Med Sci.

[30] Gafner S, Bastiaenen CHG, Terrier P, Punt I, Ferrari S, Gold G, de Bie R, Allet L (2017). Evaluation of hip abductor and adductor strength in the elderly: A reliability study. Eur Rev Aging Phys Act.

[31] Widler KS, Glatthorn JF, Bizzini M, Impellizzeri FM, Munzinger U, Leunig M, Maffiuletti NA (2009). Assessment of hip abductor muscle strength. A validity and reliability study. J Bone Joint Surg Am.

[32] Ryan AS, Buscemi A, Forrester L, Hafer-Macko CE, Ivey FM (2011). Atrophy and intramuscular fat in specific muscles of the thigh: Associated weakness and hyperinsulinemia in stroke survivors. Neurorehabil Neural Repair.

[33] Greene BR, Foran TG, McGrath D, Doheny EP, Burns A, Caulfield B (2012). A comparison of algorithms for body-worn sensor-based spatiotemporal gait parameters to the GAITRite electronic walkway. J Appl Biomech.

[34] Fletcher GF, Ades PA, Kligfield P, Arena R, Balady GJ, Bittner VA, Coke LA, Fleg JL, Forman DE, Gerber TC, Gulati M, Madan K, Rhodes J, Thompson PD, Williams MA, American Heart Association Exercise‚ Cardiac Rehabilitation‚Prevention Committee of the Council on Clinical Cardiology‚ Council on Nutrition‚ Physical ActivityMetabolism‚ Council on CardiovascularStroke Nursing‚Council on EpidemiologyPrevention (2013). Exercise standards for testing and training: A scientific statement from the American Heart Association. Circulation.

[35] Langeard A, Bigot L, Chastan N, Gauthier A (2017). Does neuromuscular electrical stimulation training of the lower limb have functional effects on the elderly?: A systematic review. Exp Gerontol.

[36] Chung Y, Kim J, Cha Y, Hwang S (2014). Therapeutic effect of functional electrical stimulation-triggered gait training corresponding gait cycle for stroke. Gait Posture.

[37] Glaviano NR, Marshall AN, Mangum LC, Hart JM, Hertel J, Russell S, Saliba S (2020). Improvements in lower-extremity function following a rehabilitation program with patterned electrical neuromuscular stimulation in females with patellofemoral pain: A randomized controlled trial. J Sport Rehabil.

[38] Kim J, Chung Y, Kim Y, Hwang S (2012). Functional electrical stimulation applied to gluteus medius and tibialis anterior corresponding gait cycle for stroke. Gait Posture.

[39] Cho M, Kim J, Chung Y, Hwang S (2015). Treadmill gait training combined with functional electrical stimulation on hip abductor and ankle dorsiflexor muscles for chronic hemiparesis. Gait Posture.

[40] Orr R, Raymond J, Fiatarone Singh M (2008). Efficacy of progressive resistance training on balance performance in older adults : A systematic review of randomized controlled trials. Sports Med.

[41] Peeters G, van Schoor NM, Cooper R, Tooth L, Kenny RA (2018). Should prevention of falls start earlier? Co-ordinated analyses of harmonised data on falls in middle-aged adults across four population-based cohort studies. PLoS One.

[42] Vespa JA, Armstrong DM, Medina L (2018). Demographic Turning Points for the United States: Population Projections for 2020 to 2060.

