# Supplementary material for: Neuromuscular Electrical Stimulation to Maximize Hip Abductor Strength and Reduce Fall Risk in Older Veterans: Protocol for a Randomized Controlled Trial
Source: JMIR Res Protoc. 2025 May 1;14:e68082. doi: 10.2196/68082 (PMC12082051; doi:10.2196/68082)
Supplement: Multimedia Appendix 1 [file resprot_v14i1e68082_app1.pdf]

SUMMARY OF DISCUSSION:
A Subcommittee of the Rehabilitation Research and Development Scientific Merit Review Board met in Plenary Session and reviewed the above proposal considering all internal and external reviews. This document summarizes the major points of the discussion concerning the proposed project. In any further development of this project, the investigator should consider carefully all the issues reflected in this Summary of Discussion as well as the more detailed comments in the individual critiques.

GENERAL COMMENTS:
The reviewers agreed that this was a very strong proposal that addresses an important and significant clinical problem, with relevance to the health and quality of life of older Veterans. The proposal included extensive and compelling preliminary data that demonstrated its feasibility and potential for success. There was agreement that the study team had been responsive to previous reviewer suggestions, and the current submission incorporates a unique, rigorous, multi-modal approach to evaluating the effects of electrical stimulation as an adjunct to balance training for older individuals.

One weakness was that selection of the 3-month time point as the primary timepoint (instead of the longer term 6- or 12-month follow-up time points) may prevent the study team from capturing the longer-term retention of intervention effects on falls risk. However, because the study team will collect up to 12-months of follow-up data, this was considered a minor weakness. There was some discussion regarding the justification for using the four-square step test as the primary outcome measure of dynamic balance, but subsequent discussion helped allay this concern. The proposed work will produce a lot of data, which is commendable, but there was some concern regarding participant burden and associated challenges with compliance with study procedures. The reviewers acknowledged that falls are a complex and multi-factorial clinical problem, so it is possible that the intervention-induced change might be relatively small or incremental. But, the proposed approach of utilizing multiple outcomes across multiple domains (muscle composition, muscle function, dynamic balance, gait performance, fall risk) was considered to be a prudent way to restore balance dysfunction and reduce fall risk.

The resubmission application was praised for being very responsive to the reviewer comments. With respect to statistical analyses, their plan related to DSMB and for accounting for statistical errors, were considered strong.

SUGGESTIONS:
No comment.

COMMENTS ON THE BUDGET:
No comment.

ADMINISTRATIVE NOTE:
Non-Veterans may only be entered into VA studies when there are insufficient Veteran patients suitable for the study (see 38 CFR 17.45, 17.92), or for studies that will generally benefit Veterans and their well-being but would not include Veterans as subjects. RR&D has noticed the investigator's plan to enroll non-Veterans. If selected for funding, a non-Veteran enrollment waiver request with sufficient justification for inclusion of non-Veterans will be required as part of JIT.

DESCRIPTION (provided by applicant):
Falls are a leading cause of injurious death and non-fatal injuries in older adults. While fall prevention is a major VA initiative, the current best fall preventions program only reduces falls by ~30% suggesting further research and interventions are needed to reduce fall risk. Decreased lower extremity muscle

mass and strength contribute to balance and mobility limitations. Our recent studies show the hip abductors have a unique role in balance and mobility function. Older adults with impaired hip abductor muscles demonstrate decreased hip abductor strength, lower balance scores, and poor stepping mechanics when recovering from a balance perturbation. Our preliminary results also show increased fatty infiltration of the hip abductor muscles may contribute to poor muscle recruitment and make changing the hip abductor muscles with a traditional strength intervention difficult. Neuromuscular electrical stimulation is one method to improve muscles in older adults, though it is not traditionally used on the hip abductors. Our preliminary results demonstrate that older adults who use neuromuscular electrical stimulation on their hip abductors, in conjunction with a balance training intervention; demonstrate larger improvements in hip abductor strength, balance, and mobility function. Our central hypothesis is that the addition of neuromuscular electrical stimulation applied to the hip abductors during a balance intervention will result in improved balance, larger improvements in muscle and mobility function, and fewer falls than a balance intervention alone. In this randomized control trial, we will determine the effects of a balance and strengthening program with and without neuromuscular electrical stimulation on fall risk and mobility (aim 1), changes in hip abductor composition, function, activation and gait variability (aim 2), and the retention of these changes at a six and twelve-month follow up (aim 3). We anticipate that this patient-oriented translational research will provide the first evidence from a large randomized control trial for the use of neuromuscular electrical stimulation applied to the primary hip abductors as an efficacious adjunct to traditional rehabilitation programs to improve balance, mobility, and muscle in older Veterans at risk for falls.

PUBLIC HEALTH RELEVANCE:

Falls are a major health concern in older Veterans. Evidence suggests that abnormalities, related to disuse, in muscles of the hips may have a major role in fall risk. This application will use novel methods of exercise to address improving the muscles of the hips as a means of reducing falls. If successful, this application will provide new practical methods to improve balance and reduce falls by improving the hip muscles in older Veterans.
